# Revealing a Novel Otubain-Like Enzyme from *Leishmania infantum* with Deubiquitinating Activity toward K48-Linked Substrate

**DOI:** 10.3389/fchem.2017.00013

**Published:** 2017-03-23

**Authors:** Clênia S. Azevedo, Bruna C. Guido, Jhonata L. Pereira, Diego O. Nolasco, Rafael Corrêa, Kelly G. Magalhães, Flávia N. Motta, Jaime M. Santana, Philippe Grellier, Izabela M. D. Bastos

**Affiliations:** ^1^Pathogen-Host Interface Laboratory, Department of Cell Biology, University of BrasiliaBrasilia, Brazil; ^2^Laboratory of Electron Microscopy, Department of Cell Biology, University of BrasiliaBrasilia, Brazil; ^3^Physics Course, Catholic University of BrasiliaBrasilia, Brazil; ^4^Programa de Pós-Graduação em Ciências Genômicas e Biotecnologia, Catholic University of BrasiliaBrasilia, Brazil; ^5^Laboratory of Immunology and Inflammation, Department of Cell Biology, University of BrasiliaBrasilia, Brazil; ^6^Faculty of Ceilandia, University of BrasíliaBrasilia, Brazil; ^7^UMR 7245 Centre Nationnal de la Recherche Scientifique, Muséum National d'Histoire Naturelle, Sorbonne UniversitésParis, France

**Keywords:** leishmania, deubiquitination, cysteine protease, molecular dynamic, site-directed mutagenesis, otubain

## Abstract

Deubiquitinating enzymes (DUBs) play an important role in regulating a variety of eukaryotic processes. In this context, exploring the role of deubiquitination in *Leishmania infantum* could be a promising alternative to search new therapeutic targets for leishmaniasis. Here we present the first characterization of a DUB from *L. infantum*, otubain (OtuLi), and its localization within parasite. The recombinant OtuLi (rOtuLi) showed improved activity on lysine 48 (K48)-linked over K63-linked tetra-ubiquitin (Ub) and site-directed mutations on amino acids close to the catalytic site (F82) or involved in Ub interaction (L265 and F182) caused structural changes as shown by molecular dynamics, resulting in a reduction or loss of enzyme activity, respectively. Furthermore, rOtuLi stimulates lipid droplet biogenesis (an inflammatory marker) and induces IL-6 and TNF-α secretion in peritoneal macrophages, both proinflammatory cytokines. Our findings suggest that OtuLi is a cytoplasmic enzyme with K48-linked substrate specificity that could play a part in proinflammatory response in stimulated murine macrophages.

## Introduction

Visceral leishmaniasis (VL), also known as kala-azar, is the systemic form of vector-borne disease leishmaniasis, mostly caused by *Leishmania infantum* and *L. donovani* that affects visceral organs and triggers general damages including hepatosplenomegaly and lymphadenopathy (Pearson and Sousa, 1996). VL can be fatal if left untreated and, due to the associated cellular immunity depression, predisposes to secondary infections (Choi and Lerner, 2001). Majority of VL cases are concentrated in Africa and South America, and ~90% of known cases in America take place in Brazil, especially in the Northeast region (Harhay et al., 2011). Although, 200,000 cases are estimated and ~20,000 deaths are assigned to VL (Alvar et al., 2012), the available pharmacological intervention faces emerging resistance and severe side effects (Kobets et al., 2012).

The establishment of *Leishmania* infection is dependent upon the success of the invasion of phagocytic cells, mainly macrophages, and dendritic cells, by promastigote forms and the survival of amastigote forms inside the cells (reviewed in Rodrigues et al., 2016). Subverting host's immune response is essential to determine the disease progression. Post-translational modifications, such as ubiquitination, seem to be a regulator of host-pathogen interactions through the modulation of immune signaling (Srivastav et al., 2012).

Ubiquitination consists of the covalent binding of ubiquitin molecules (Ub) to a protein substrate in order to regulate signaling pathways, either taking these target proteins for degradation by the 26S proteasome or modifying their activity or localization (Husnjak and Dikic, 2012). Ubiquitin linking to a substrate can be realized through any of the 7 lysines present in the ubiquitin molecule (K6, K11, K27, K29, K33, K48, and K63) or the N-terminal methionine residue. Depending upon linkage, the target could fate different outcomes in the cell (Komander and Rape, 2012). Polyubiquitinated targets on K48 represent the vast majority in eukaryotic cells and they are directed to the 26S proteasome for degradation (Hershko and Ciechanover, 1998). Polyubiquitinated chains on K63 are involved in several non-degradative cellular processes such as DNA repair (Doil et al., 2009; Stewart et al., 2009), gene transcription (Wang et al., 2001; Xu et al., 2009), and innate immune responses (Gack et al., 2007). Insights concerning the role of polyubiquitination on other lysines is still advancing (reviewed in Swatek and Komander, 2016; Yau and Rape, 2016).

This post-translational modification can be reversed by the deconjugation of the Ub molecule or chain by specialized proteases called deubiquitinating enzymes (DUBs) capable of regenerating free ubiquitin molecules. DUBs play an important role in the regulation of various processes, such as tumor progression, immune regulation, and neurodegeneration (Nijman et al., 2005). DUBs can be divided into five families, from which 4 are cysteine-proteases: ubiquitin carboxyl-terminal hydrolase (UCHs), ubiquitin-specific protease (USPs), ovarian tumor-related proteases (OTUs), Machado-Joseph disease proteases (MJDs), and one JAB1/MPN/MOV34 metallo-protease (JAMMs) (Komander et al., 2009). These enzymes have a substrate specificity that is conferred by their protein interaction domains, the nature of the ubiquitin chain linkage and their subcellular localization (Wertz et al., 2004; Nijman et al., 2005; Kayagaki et al., 2007).

Otubain is a protease family member related to OTUs, the second largest DUB family in mammals, first described in *Drosophila melanogaster* (King and Storto, 1988) and related to the regulation of T cell anergy by interacting with the E3 ligase GRAIL promoting activation of T cells in mice (Soares et al., 2004). Proteases containing the OTU domain seem to have appeared in eukaryotes' evolution by acquisition from intracellular pathogens, indicating that the presence of these proteases may be related to host-pathogen interaction (Makarova et al., 2000). The *Chlamydia caviae* OTU (ChlaOTU) has deubiquitinating activity and proved to be an effector type III secretion protein. ChlaOTU is able to deubiquitinate proteins on the entrance site of *C. caviae* into the cell and also binds to NDP52, a host protein important to the invasion and to bacterial growth inside the host cell (Furtado et al., 2013). Regarding parasites, the first active DUBs were identified in *Plasmodium falciparum* (Artavanis-Tsakonas et al., 2006) and *Toxoplasma gondii* (Frickel et al., 2007). Transcriptional analyses of MJD and OTU transcripts from *Schistosoma mansoni* demonstrated differential regulation throughout the parasite cycle (Pereira et al., 2015). Genetic analyses showed that the parasite *P. chabaudi* resistant to chloroquine and artesunate have a mutation in a DUB (Hunt et al., 2007). Otubain from *Cryptosporidium parvum* (CpOTU) has been characterized showing that it is more expressed in the oocyst stage of the parasite (Ju et al., 2014).

Since OTU family enzymes seem to be involved in the regulation of different cellular processes, including immunological pathways regulation, and considering that the establishment of *Leishmania* in its host depends on host immune response modulation, it seems promising to investigate the relevance of deubiquitination in *Leishmania* infection. In this work, we first characterized an otubain-like enzyme presented in the cytoplasm of *L. infantum* promastigotes with preferential K48-linked deubiquitinating activity *in vitro*. In this sense, we generated three recombinant OtuLi variants carrying point mutations on OTU domain and on a hydrophobic surface important for interaction with ubiquitin residues (Nanao et al., 2004). We showed that recombinant OtuLi cleaves K48-linked tetra-ubiquitin and the F82 besides the catalytic C81 seems critical to its enzymatic activity. K63-linked tetra-ubiquitin is also substrate for rOtuLi, although such activity is less expressive and completely abolished when a single amino acid present in the Ub-interaction motif is changed (F182). Altogether, these results compose the first study concerning the deubiquitinating enzymes in *Leishmania* and represent a prelude in order to understand the deubiquitination process in kinetoplastids.

## Materials and methods

### Parasites

*L. infantum* promastigotes were maintained in Schneider's Insect medium (Sigma Aldrich) supplemented with 20% (v/v) heat-inactivated fetal bovine serum (FBS) and 100 μg/mL gentamycin sulfate at 28°C.

### Recombinant OtuLi expression in *E. coli*

Plasmids (pET19-b) containing the WT *otuli* or the sequence carrying the site-directed mutations that generated the F182S, F182S/L265P, and F82S/F182S/L265P variants (T545C, T545C/T794C, and T245C/T545C/T794C as nucleotide sequence respectively) were synthesized by GenScript. The constructions were expressed in BL21 (DE3) *E. coli* and produced the C-terminal His-tagged OtuLi under induction by 0.1 mM IPTG at 18°C for 5 or 16 h (F82S/F182S/L265P variant). The cells were lysed with BugBuster™ (Merck) and the supernatant was applied into the His-Bind® column (Novagen), washed with increasing concentration of imidazole and harvested with elution buffer (500 mM NaCl, 20 mM Tris-HCl, 200 mM imidazole, pH 7.5). After enzyme purification, WT rOtuLi and the 3 variants were dialyzed and concentrated with 25 mM Tris-HCl, pH 7.5. Purified enzymes was stored in 50% glycerol at −20°C and analyzed by SDS/PAGE (12% polyacrylamide gel) under reducing conditions with coomassie blue or silver staining.

### Antibody production

The purified WT rOtuLi (5 μg) in Freund's complete adjuvant was used to immunize male BALB/c mice, followed by three biweekly immunizations in Freund's incomplete adjuvant. Immune sera were collected 5 days after the last immunization, diluted in glycerol (1:1) and analyzed by western blotting to evaluate the specific antibody production against the protein extract from *L. infantum* and the purified recombinant protein.

### Western blotting analysis

*L*. *infantum* promastigotes previously washed with PBS were submitted to the freezing/thawing process repeatedly in order to lyse cells and to obtain the soluble (SE) and insoluble (IE) protein extract. The proteins were transferred to a nitrocellulose membrane, blocked for unspecific antibody's interactions with TBS containing 5% milk followed by anti-OtuLi antibody (1:100) incubation and goat anti-mouse IgG horseradish peroxidase conjugated (1:30,000). Blotting analyses were developed using the ECL™ Prime Western blotting detection reagent (GE Healthcare) and performed in ImageQuant LAS 4,000 (GE HealthCare).

### Recombinant OtuLi deubiquitination and inhibition assays

The enzyme reaction was performed with 250 ng of rOtuLi and 250 ng of K48- or K63-linked tetra-ubiquitin in 25 mM Tris pH 7.5 or 25 mM MES pH 5.5 for 5 h. For inhibition profile, 100 μM N-Ethylmaleimide (NEM) and 100 μM *trans*-Epoxysuccinyl-L-leucylamido(4-guanidino)butane (E-64) were incubated with rOtuLi for 30 min before addition of tetra-ubiquitin. Reactions were interrupted by addition of sample buffer (62.5 mM Tris–HCl pH 6.8, 2% m/v SDS, 5% v/v β-mercaptoethanol, 0.08% m/v bromophenol blue and 30% v/v glycerol) and analyzed by 12% SDS/PAGE, followed by silver staining.

### Homology modeling

The search for template was made in PDB—Protein Data Bank (www.rcsb.org/pdb) and the crystal structure of human otubain (2ZFY) (Edelmann et al., 2009) was chosen. The alignment of the target sequence (OtuLi) with the template (HsOtu1) was made using the T-Coffee software resulting in 32% identity and 44% similarity. Then, a model was constructed using the Swiss Model Server and the structural figures were generated with PyMOL system. The OtuLi model was validated using PROCHECK software (Laskowski et al., 1996).

### Molecular dynamics

The simulated ensembles are comprised of the macromolecule otubain from *L. infantum*, in both WT and inactive (F82S/F182S/L265P) forms, immersed in TIP3P water molecules (Berendsen et al., 1981). For each conformation, all-atom models of the non-bonded conformation and the OtuLi-Ubiquitin complex were constructed in GROMACS 4.6.7 using the CHARMM22 force field (Lazaridis and Karplus, 1999; Brooks et al., 2009; Pronk et al., 2013). Each protein was amidated at its C-terminus. All four different conformations were contained using periodic boundary conditions with unit cells corresponding to dodecahedral boxes with dimensions that enable adequate sampling without added complications arising from the proteins directly interacting with their mirror images. The four systems were then individually solvated with TIP3P water (wild non-bonded: 8,365 molecules TIP3P; wild OtuLi-Ubiquitin complex: 19,117 molecules TIP3P; Inactive non-bonded: 8,208 molecules TIP3P; inactive OtuLi-Ubiquitin complex: 20,124 molecules TIP3P), and salt ions were added to achieve electro-neutrality at a final salt concentration of 1.5M. After generating the solvated structures, the system was energy minimized using steepest descent until the change in potential energy between time-steps converged to machine precision. After energy minimization, the system underwent two phases of equilibration by using the Berendsen pressure and temperature coupling methods; it was first equilibrated with a time-step of 2 fs for 10 ps under constant pressure, and then was equilibrated with a time-step of 2 fs for 10 ps under constant pressure (Berendsen et al., 1984). Finally, the system underwent a 2 ns simulation of position restraint, in which the protein was restrained and the solvent molecules were relaxed through an energy minimization step under constant temperature by using the Berendsen temperature coupling method. All simulations were run under constant pressure and temperature (NPT). We performed a total of 200 ns of molecular dynamics simulations to sample the largest possible conformational space. During the production runs, the LINCS algorithm was used to constrain the lengths of bonds involving Hydrogen atoms, which vibrate faster than our simulation time-step of 2 fs (Hess et al., 1997), and the SETTLE algorithm was used to constrain the bond lengths within water molecules (Miyamoto and Kollman, 1992). Electrostatic calculations were performed using the Particle Mesh Ewald (PME) method with a cut-off radius of 1.4 nm (Darden et al., 1993). The same radius value cut-off was also used in the van der Waals calculations. The non-bonded list of each atom was updated every 10 simulation-steps. All simulations were performed using GROMACS 4.6.7 with the CHARMM22 force field with a machine temperature of 300 K.

### *In vitro* stimulation of peritoneal macrophages by rOtuLi

Murine peritoneal macrophages (10^6^ cells/mL) from six C57BL/6 mice were harvested by peritoneal lavage and adhered for 16 h in RPMI medium supplemented with 2% FBS and non-adherent macrophages were removed by washing with PBS. Cells were stimulated with 1 μg of rOtuLi or the boiled denatured enzyme for 24 and 48 h at 37°C and 5% CO_2_ atmosphere.

### Lipid droplet biogenesis analysis

Peritoneal macrophages seeded in 24-well plate coverslips stimulated or not with rOtuLi were fixed in 3.7% (v/v) formaldehyde diluted in PBS. Then, the coverslips were washed three times with ultrapure water and incubated with Propylene Glycol (PEG) for 15 min. Subsequently, the coverslips were incubated with 0.5% Oil Red for 30 min and with 60% PEG for 1 min. After three washes, cells were stained with hematoxylin solution for 5 s. Finally, the slides were mounted with Aqua Poly/Mount medium (Vector). Lipid droplet biogenesis was assessed by quantification of lipid droplet structures on 50 consecutive cells by bright field microscopy.

### Quantification of cytokines

The supernatants of the peritoneal macrophages stimulation assay with rOtuLi were collected at 24 and 48 h. IL-6 and TNF-α cytokines were measured by ELISA, using a commercial kit (eBioscience). The assay was performed following manufacturer's instructions and cytokine levels were demonstrated in absolute values (pg/mL).

### Fluorescence microscopy

For mitochondrion labeling, *L. infantum* promastigotes were washed with PBS, incubated in 80 nM MitoTracker Red CMXRos (Molecular Probes) for 1 h. Then, parasites were washed with PBS and immunofluorescence assay was conducted by fixing them with 3.7% paraformaldehyde with subsequent permeabilization with 0.1% Triton X-100 and then spread onto a poly-L-lysine coated glass slide. For OtuLi labeling, parasites were incubated with the primary antibody anti-OtuLi (1:100) for 2 h. For PDI labeling, an anti-PDI antibody from *Gallus gallus* was used in a 1:500 dilution for 2 h. After washing with PBS, parasites were incubated with one of the following antibodies: goat *anti–mouse* IgG conjugated with Alexa Fluor 488 (1:500) for OtuLi labeling, goat anti-rabbit IgG conjugated with Alexa Fluor 568 (1:300) for PDI labeling both for 2 h. For DNA staining, 100 ng/mL DAPI were used for 10 min and the slides were mounted with ProLong® Gold antifade (Invitrogen) and observed under a Nikon Eclipse TE 300 DV inverted microscope using appropriate fluorescence emission filters at room temperature. Data acquisition and processing were performed with MetaMorph and ImageJ software, respectively.

### Ethics statement

This study was carried out in accordance with institutional guidelines. The protocol was approved by Comissão de Ética de Uso Animal from the University of Brasilia, No. 27764/2016.

### Statistical analysis

The graphics were generated using the Software GraphPad Prism. The results of lipid bodies and cytokines quantification were expressed as mean ± standard error of the mean (SEM) and statistically analyzed with analysis of variance (ANOVA) followed by *t*-Student test. The p significance level was broken down in the results.

## Results

### OtuLi sequence analyses and expression pattern

Analysis of the *L. infantum* genome was performed using the Database of Ubiquitinating and Deubiquitinating Enzymes (http://www.DUDE-db.org/) (Hutchins et al., 2013), and provided 27 predicted proteins of which 16 are from the USP family, 5 from the JAMM family, 4 from the OTU family, and 2 from the UCH family. There were no predicted genes coding to proteases belonging to the MJD family. The otubain cysteine peptidase herein named OtuLi (XP_001464809.1) was selected among the predicted OTU enzymes for further characterization based on its low amino acids identity compared to the human otubain-1 (33%; AAO27702.1) and otubain-2 (26%; AAO27703.1).

**Figure 1:**
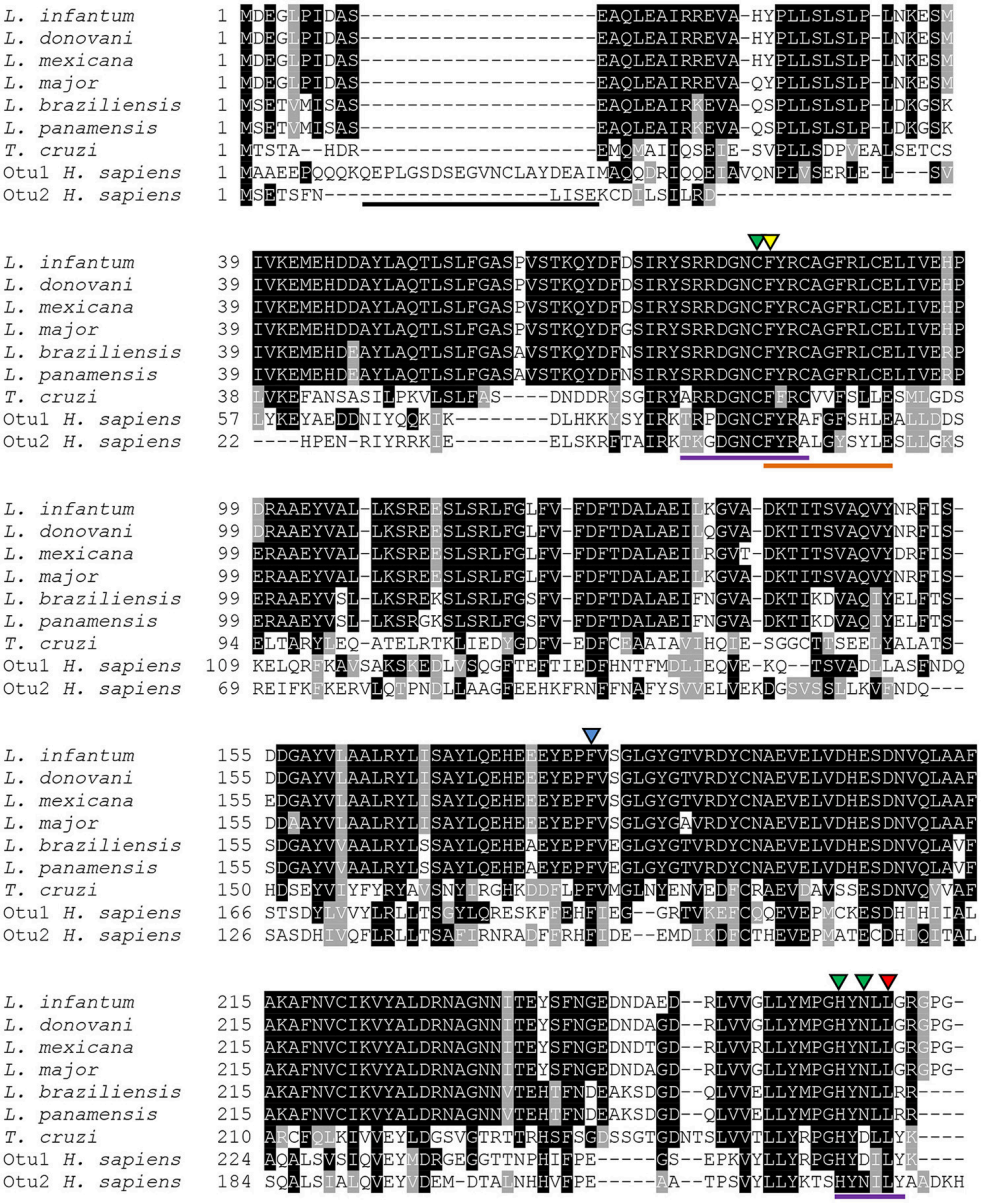
**Alignment of otubain sequences from different organisms**. Amino acid sequences available at NCBI from *Leishmania infantum* (XP_001464809.1), *L. donovani* (CBZ33300.1), *L. mexicana* (XP_003874007.1), *L. major* (CAJ04142.1), *L. braziliensis* (XP_001563920.1), *L. panamensis* (AIN97175.1), *Trypanosoma cruzi* (XP_816450.1), otubain-1 from *Homo sapiens* (NP_060140.2) and otubain-2 from *H. sapiens* (NP_075601.1) were aligned using the T-Coffee software. Lines represent the extended N-terminal from HsOtu1 (black), the OTU domain (purple) and the ubiquitin-interaction motif (orange). The green triangles correspond to the catalytic triad. The conserved amino acids chosen to be replaced are F82 (yellow), F182 (blue), and L265 (red).

The *otuli* gene has an open reading frame of 813 bp that codifies a protein of 270 amino acids corresponding to a predicted molecular weight of 30.3 kDa. The catalytic triad consists of the Cys81, His261, and Asn263 residues with an ubiquitin interaction motif (UIM) preserved. The ubiquitin-associated (UBA) domain and the nuclear localization signal which are commonly conserved in OTU family proteins and an extended N-terminal present in HsOtu1 are absent (Figure 1). Otubain is conserved between the subgenus *Leishmania*, mainly with *L. donovani* (99%) and *L. major* (98%). Regarding its counterpart in *Trypanosoma cruzi*, OtuLi shows only 39% identity.

**Figure 2:**
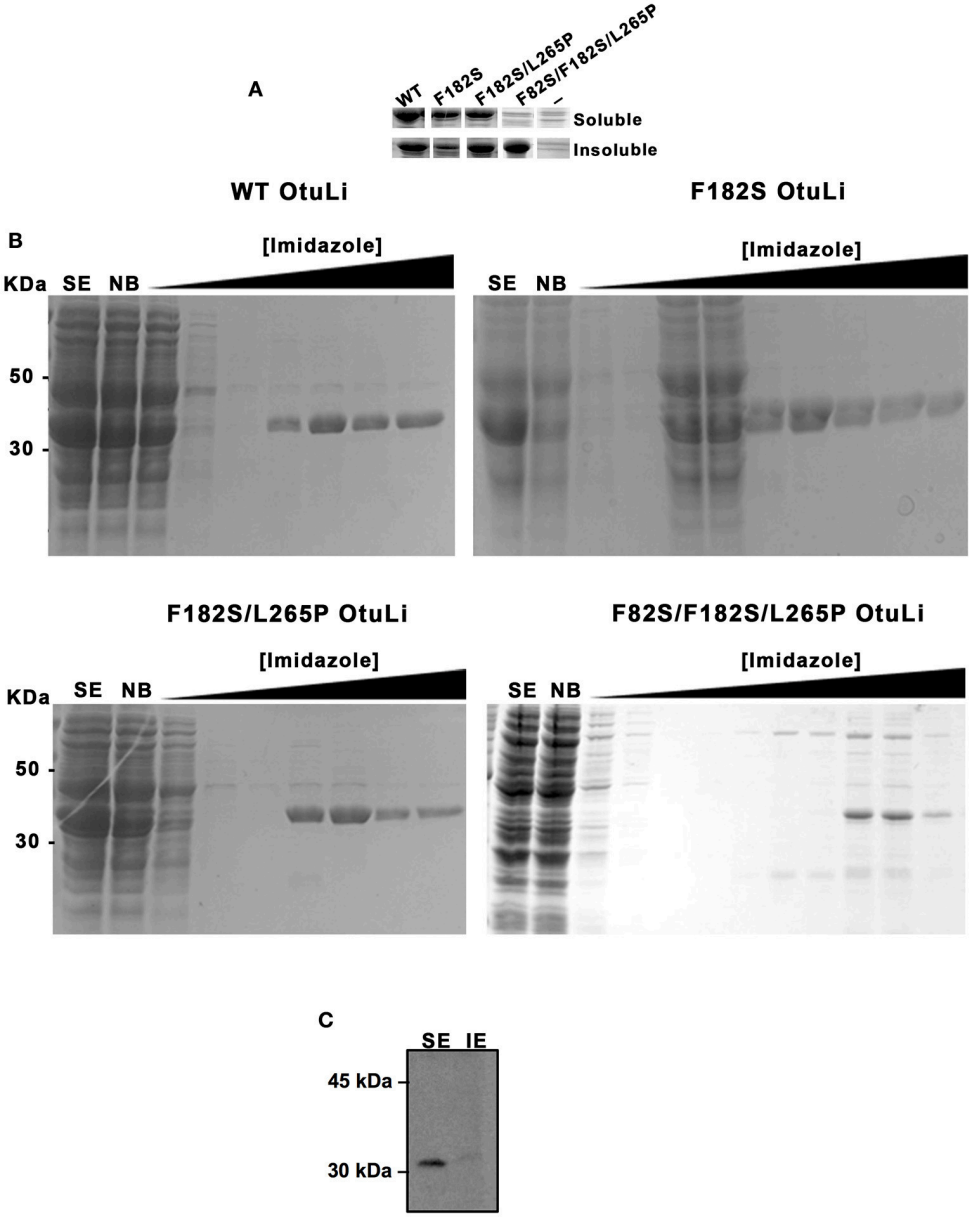
**Expression of OtuLi in bacteria and identification of the native enzyme in promastigote forms. (A)** Expression of the recombinant enzymes in *E. coli* BL21 (DE3) in soluble or insoluble extracts was compared to the expression of empty pET-19b (−) analyzed by coomassie blue staining. **(B)** Purification of the WT rOtuLi and its variants by nickel affinity chromatography. Non-bounded proteins (NB), wash fractions obtained with increasing concentration of imidazole and elution with 200 mM (or 400 mM for F82S/F182S/L265P variant) imidazole were analyzed by coomassie blue staining. **(C)** Confirmation of OtuLi in soluble extract (SE) from promastigote forms by western blotting.

Prior the enzymatic characterization of OtuLi, we obtained its recombinant enzyme (rOtuLi) by expression in prokaryotic system using vector pET19-b, which fused the enzyme to a histidine tail used for nickel-agarose purification. In order to better understand the catalytic mechanism, site-directed mutations were introduced into *otuli* sequence generating a single (F182S), a double (F182S/L265P), and a triple (F82S/F182S/L265P) variant. These mutations affected conserved amino acids that are located next to the Cys residue from the active site (F82), inside OTU domain (L265), or part of a hydrophobic surface (F182) important to the interaction with the ubiquitin molecule (Nanao et al., 2004). Variants were also obtained in prokaryotic system using the same expression vector. WT OtuLi, F182S and F182S/L265P variants were produced in abundance as both soluble and insoluble forms in BL21 (DE3) *E. coli*. However, the F82S/F182S/L265P variant was produced in small amount as soluble enzyme (Figure 2A). Nevertheless, WT and the 3 variants were successfully obtained as purified enzymes and used to perform enzymatic assays (Figure 2B). Native OtuLi was identified in soluble extract from promastigote forms of *L. infantum* using the polyclonal antibody against the WT rOtuLi produced in mice (Figure 2C).

### Enzymatic activity and comparative molecular modeling of WT OtuLi and variants

**Figure 3:**
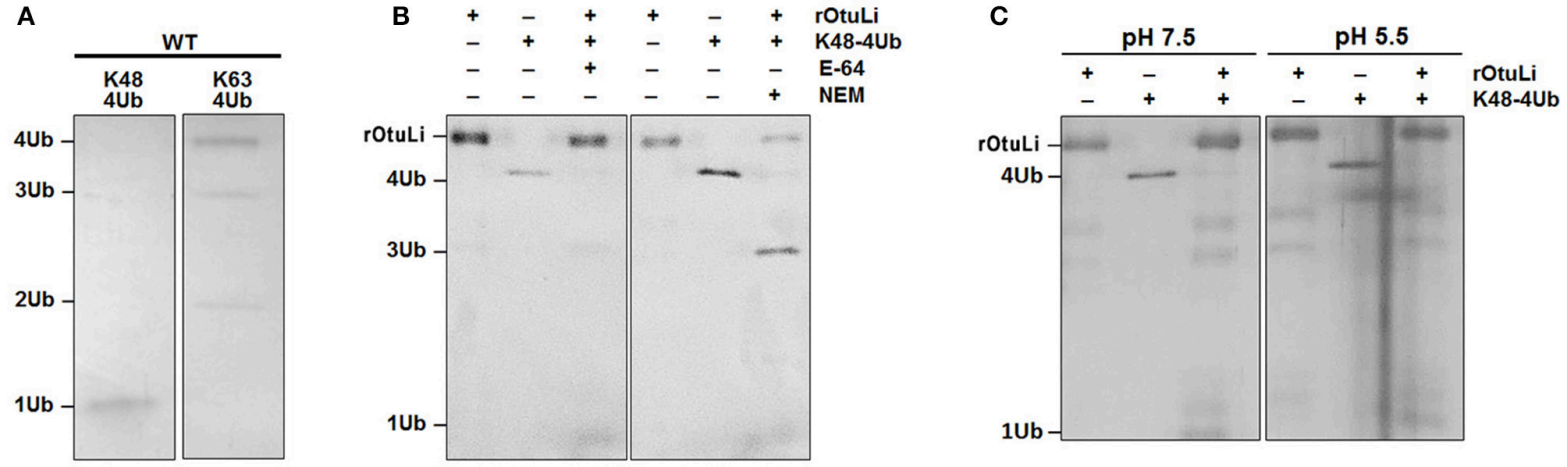
**Enzymatic activity characterization of rOtuLi. (A)** Enzymatic activity of rOtuLi against K48-4Ub or K63-4Ub substrates in 25 mM Tris pH 7.5. **(B)** Inhibition of rOtuLi in the presence of classic inhibitors in 25 mM Tris pH 7.5. **(C)** Enzymatic activity of rOtuLi with K48-4Ub in 25 mM MES pH 6.5. All reactions were carried out for 5 h at 25°C, stopped by adding sample buffer and analyzed by silver staining.

The deubiquitinating activity was assessed by rOtuLi capability of cleaving K48- and K63-linked tetra-ubiquitin (4Ub) substrates at pH 7.5. rOtuLi cleaves preferentially K48-4Ub over K63-4Ub, as indicated by the complete cleavage to monoubiquitin (1Ub) in the K48-4Ub reaction and the partial activity of OtuLi against K63-4Ub with hydrolysis to tri-ubiquitin and di-ubiquitin (Figure 3A). Enzymatic activity was also assessed with fluorescent substrates such as Ub-AMC and Z-LRGG-AMC but rOtuLi was inactive (data not shown).

Considering the rOtuLi specifity for K48-4Ub, the inhibition pattern of the enzyme was assessed with this same substrate. Being OtuLi classified as a cysteine peptidase, the standard cysteine peptidase inhibitors E-64 and NEM were tested. Surprisingly, rOtuLi was not inhibited by E-64 and inhibited in a moderate manner by NEM, as we could see an intense band corresponding to 3Ub (Figure 3B). We also assessed the enzymatic activity in acidic environment and rOtuLi remained active at pH 5.5 (Figure 3C).

**Figure 4:**
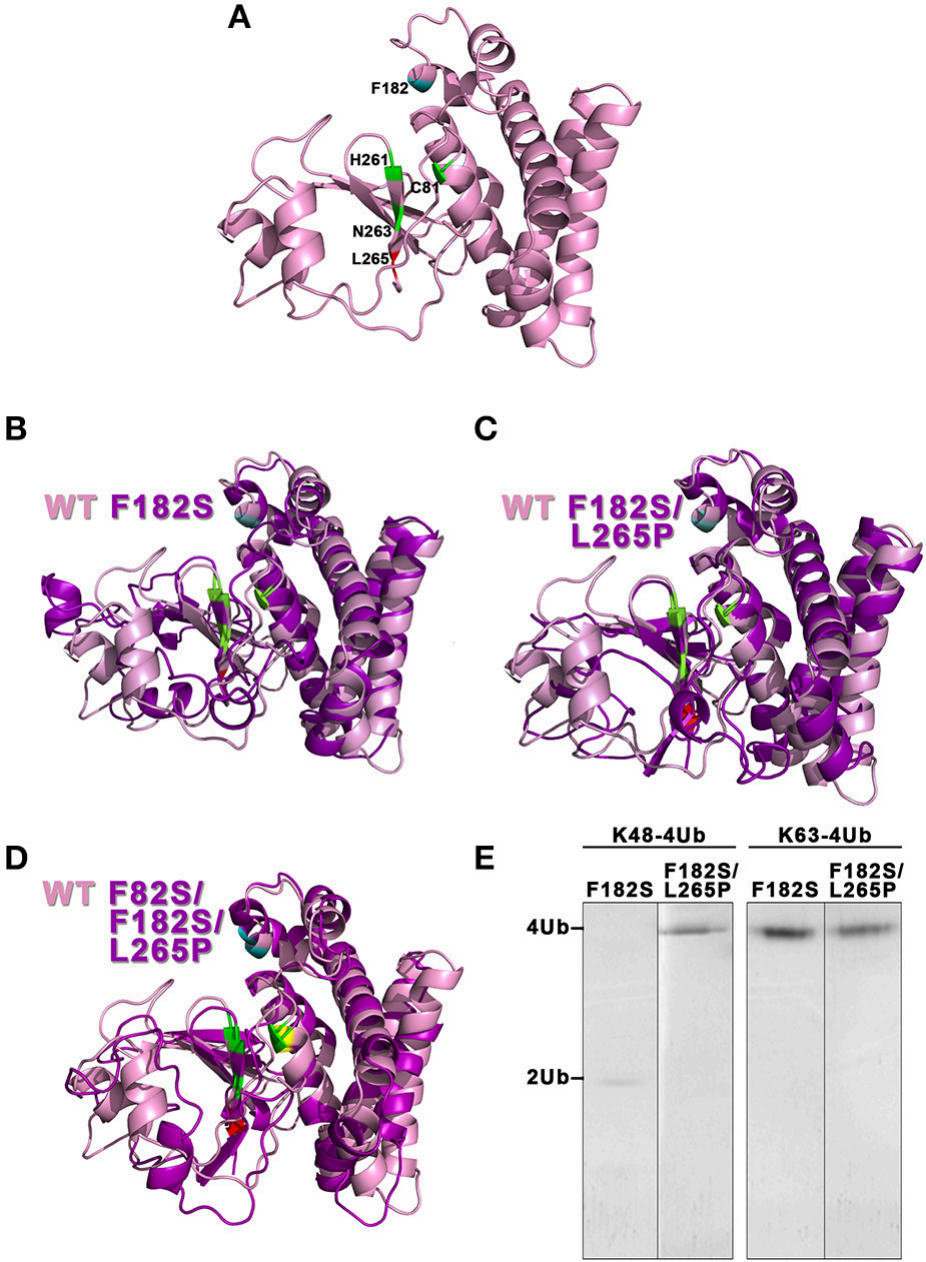
**Evaluation of the molecular models from OtuLi and its variants**. The three-dimensional models of WT OtuLi **(A)** and the overlapped models of F182S **(B)**, F182S/L265P **(C)**, and F82S/F182S/L265P **(D)** with WT OtuLi. WT OtuLi (pink) and its variants (magenta) models show the catalytic triad (C81, H261, and N263) in green and the amino acids F82S (yellow), F182S (blue), and L265P (red). **(E)** Enzymatic activity of the variants against K48- or K63-4Ub in 25 mM Tris pH 7.5.

**Figure 5:**
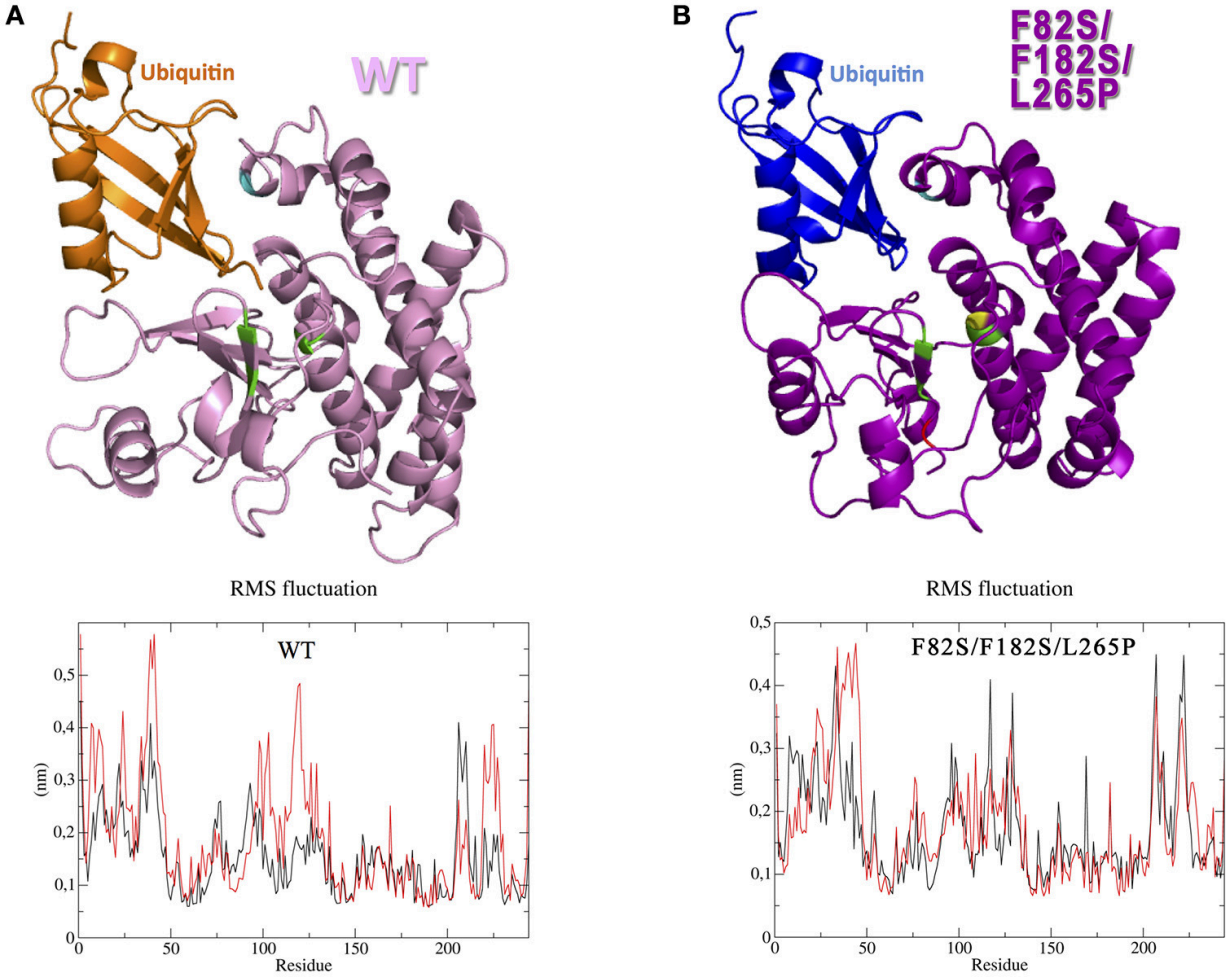
**Interaction between OtuLi and ubiquitin**. Simulated ensembles of OtuLi and an ubiquitin molecule, using WT enzyme **(A)** and F82S/F182S/L265P variant **(B)**. Amino acids mobility in WT OtuLi (**A**, bottom) and F82S/F182S/L265P OtuLi (**B**, bottom) models considering their free (black) and Ub-complexed (red) forms.

According to the homology modeling, the OtuLi model comprises 10 α-helixes and 4 β-strands that altogether constitute the β-sheet responsible for stabilizing the core of the protein. This portion provides the catalytic triad formed by C81, H261, and N263 residues (Figure 4A). There were significant changes between the WT OtuLi and the variants' models, mostly with F82S/F182S/L265P as shown by the overlapped models (Figures 4B–D) and by the RMSD values. Comparing the RMSD values, it was observed a difference of 1,512 Å (WT and F182S), 1,690 Å (WT and F182S/L265P), whereas the main difference (2,484 Å) was between WT and F82S/F182S/L265P. The F182S variant remains active against K48-linked substrate. However, the addition of L265P mutation (F182S/L265P) abolishes the enzyme activity against K48-4Ub, since no cleavage was seen with this mutant (Figure 4E, left). Regarding the enzymatic activity against K63-4Ub substrate, the F182S mutation was enough to confer inactivity to OtuLi (Figure 4E, right), implying that F182 residue is crucial to OtuLi activity against K63-linked substrates. Molecular dynamics simulations of the OtuLi-Ub complex showed an elevated degree of flexibility for some residues from the WT enzyme when compared to its free form. This flexibility is observed when we compare the root mean square fluctuation per residue of both the free and complex forms. Moreover, the same was not observed on F82S/F182S/L265P variant and Ub complex simulation (Figure 5). This lack of flexibility may explain the large amount of inclusion bodies observed on F82S/F182S/L265P variant produced in bacteria (Figure 2A), besides its inactivity against both substrates (data not shown).

### OtuLi is a cytoplasmic enzyme and induces an inflammatory response *in vitro*

**Figure 6:**
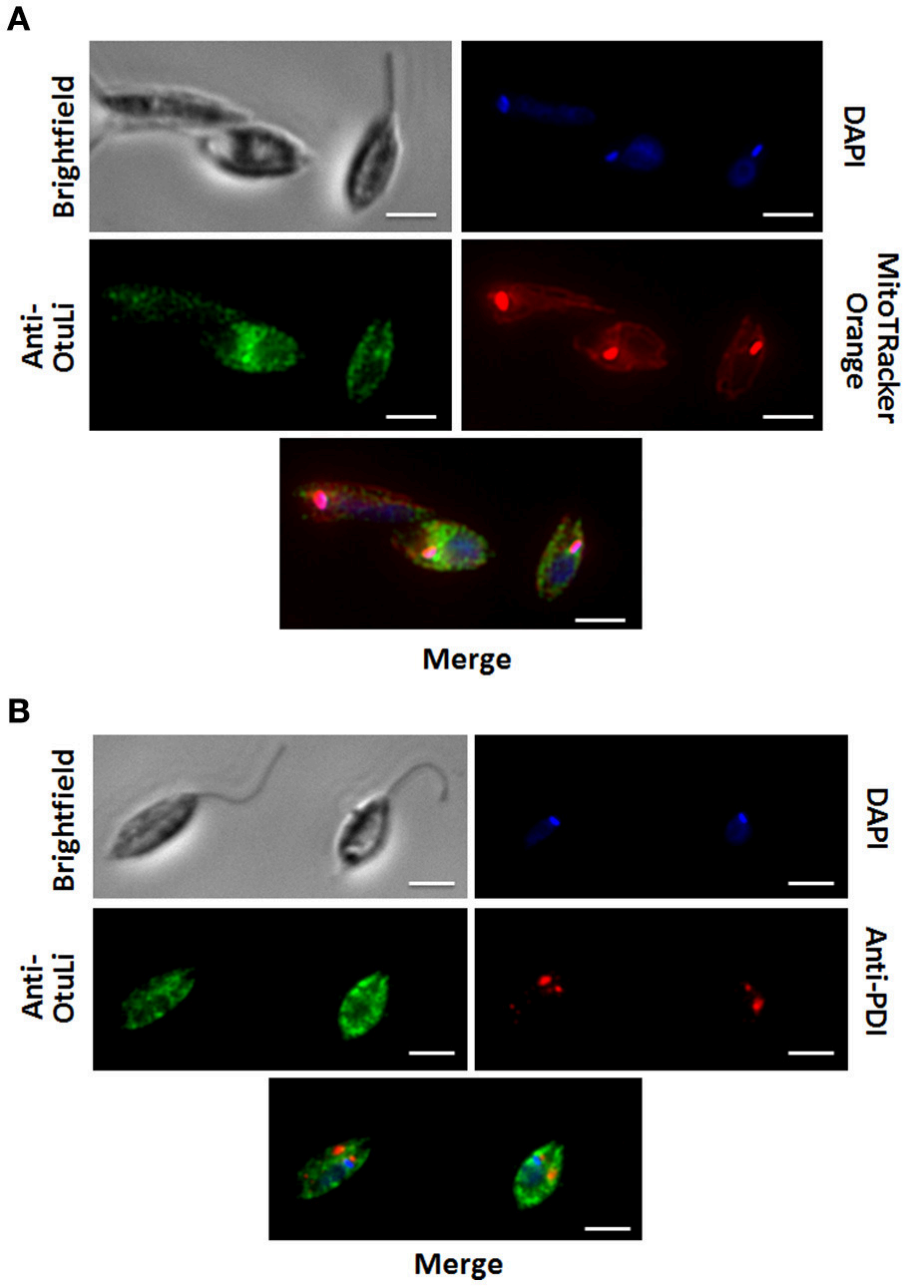
**OtuLi cellular localization**. Cytolocalization of OtuLi, mitochondrion probed with Mitotracker Orange **(A)** and PDI **(B)**. Genomic DNA and kinetoplast (blue) were labeled by DAPI. Bars: 5 μm.

**Figure 7:**
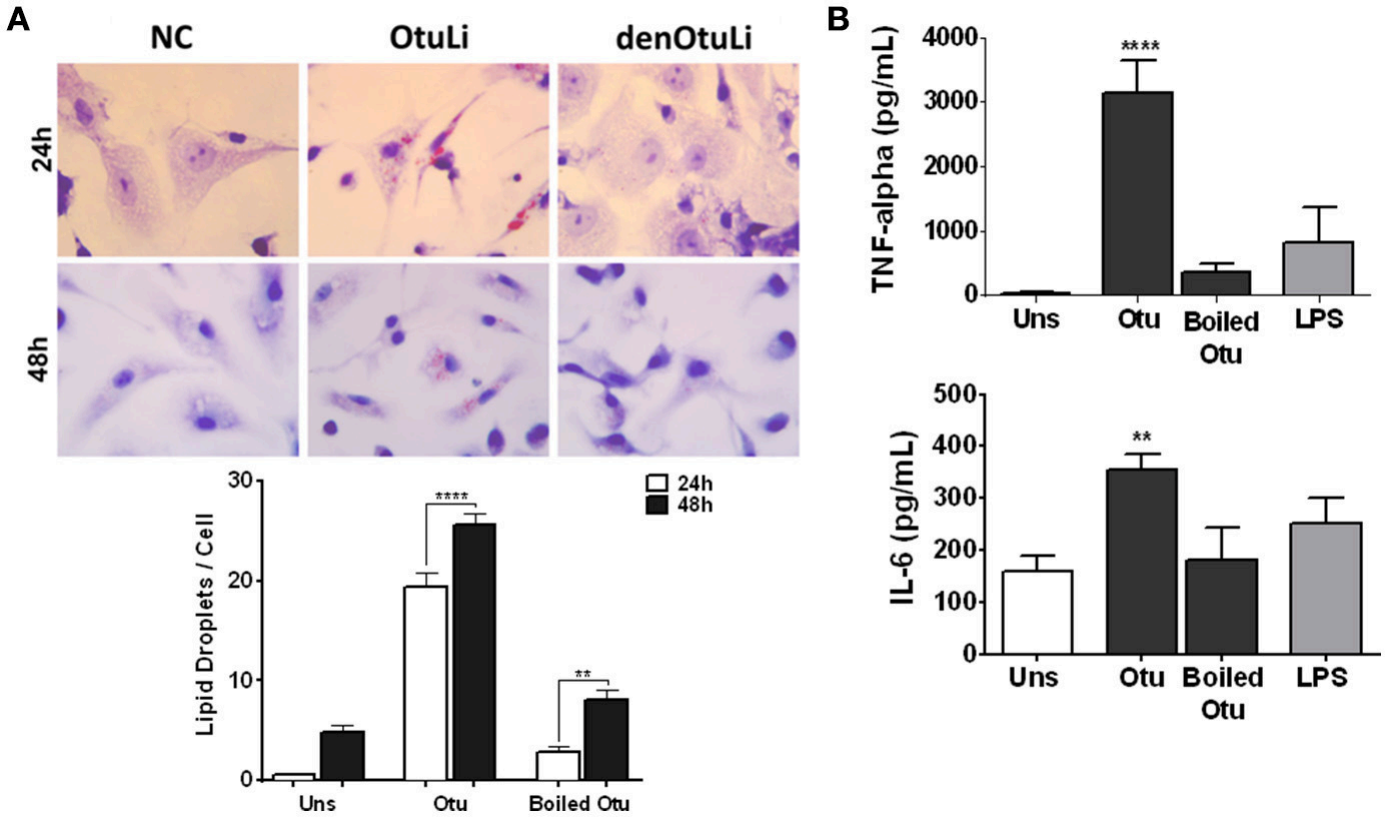
**Immunomodulatory profile of rOtuLi in murine macrophages. (A)** Peritoneal macrophages from C57BL/6 mice incubated with boiled rOtuLi (denOtuLi/boiled Otu) or rOtuLi (Otu) for 24 and 48 h were observed by light microscopy at 50 cells separately. Original magnification × 100 (^**^p < 0.01, ^****^p < 0.0001). **(B)** Cytokine production from stimulated or unstimulated (Uns) macrophages for TNF-α and IL-6 production levels were assessed by ELISA (^**^p < 0.01, ^****^p < 0.0001).

In order to investigate the localization of OtuLi in promastigotes, a mitochondrion probe, or the Protein Disulfide-Isomerase (PDI) antibody (Florent et al., 2000) were employed to label mitochondrion and endoplasmic reticulum, respectively. OtuLi is presented in small vesicles along the cytoplasm of the cell, although an intense fluorescence can be observed near kinetoplast. There was no co-localization with mitochondrion or PDI (Figure 6). To examine whether rOtuLi could have an immunomodulatory effect, murine peritoneal macrophages were incubated with the enzyme and analyzed after 24 and 48 h of stimulation. rOtuLi triggered prominent lipid droplet biogenesis (a structural marker of inflammation) in macrophages compared both to unstimulated cells and macrophages stimulated with denatured rOtuLi, which presented three-fold less amount of lipid droplet after 48 h (Figure 7A). In addition, rOtuLi was able of triggering significant TNF-α and IL-6 secretion in macrophages compared to unstimulated cells after 24 h (Figure 7B).

## Discussion

Here we paved the way for further studies about deubiquitination in trypanosomatids, especially *Leishmania*, by characterizing the otubain-like enzyme from *L. infantum*. Considering biochemical aspects, rOtuLi was able to cleave both K48- and K63-linked substrates, although its enzymatic activity is less pronounced on the last one. Otubains are classified as cysteine proteases and rOtuLi was inhibited by NEM, however the described otubain from *C. parvum* (CpOTU) (Ju et al., 2014) and rOtuLi showed activity with E-64, a well-defined inhibitor for cysteine proteases. Whereas, the majority of cysteine proteases have optimal activity in acidic pH (Turk et al., 1995), it was investigated whether rOtuLi was active at pH 5.5. In fact, rOtuLi is active in more acidic pH in a similar manner to the activity at neutral pH, unlike CpOTU that showed instability in acid pH and its optimal enzymatic activity is at pH 7.5 (Ju et al., 2014).

The HsOtu1 and HsOtu2 structures reveal that the active site is formed by an α-helix and β-sheet lobes centering the catalytic C91/H265/D267 or C51/H224/N226, respectively, where the conformation of H265 in HsOtu1 and its distance to the C91 and D267 is incompatible with catalysis indicating a singularity face to other cysteine proteases (Edelmann et al., 2009). This steric incompatibility of the catalytic site seems to be reversed with the structural rearrangement of the molecule when the ubiquitin binds to the ubiquitin interaction motif, also present in OtuLi, which leads to Ub chain cleavage. In fact, as shown by our molecular dynamics assays, the substrate seems to play an important role over the mobility of specific amino acids, therefore contributing to the WT OtuLi structure rearrangement for proper substrate accommodation in the catalytic pocket, which was not observed in the inactive F82S/F182S/L265P variant.

Furthermore, considering the sequence analysis, the lack of an N-terminal extension allows the interaction with ubiquitin in a more permissive way, which could explain the fact that rOtuLi cleaves both ubiquitin chains connected to K48 and K63, as well as HsOtu2 (Altun et al., 2015). However, this flexibility was not sufficient for enabling the cleavage of Ub-AMC by rOtuLi while HsOtu2 is capable of cleaving Ub-AMC because the AMC can be properly accommodated in the catalytic pocket where G47 seems to be relevant for this process (Edelmann et al., 2009). In rOtuLi the correspondent residue is R77 and P87 in HsOtu1 (also unable to cleave Ub-AMC) that promotes a steric hindrance in the catalytic core that compromises both interaction and cleavage of this substrate (Edelmann et al., 2009).

To describe the role of critical amino acids, three different variants carrying point mutations were also studied. The F182 residue in OtuLi is a conserved amino acid and is present in a hydrophobic surface of HsOtu2 which is exposed and form a pocket for interaction with ubiquitin residues (Nanao et al., 2004). Thus, the purpose was to evaluate if the switch of the F182 for a hydrophilic amino acid (S182) would impair enzymatic activity, but it was not enough to cause the loss of OtuLi activity against K48-4Ub, although F182 seems to be important to the cleavage of K63-4Ub once it was not possible to detect activity with this latter substrate. Upon the introduction of a bulky amino acid (P265) next to a conserved residue, part of the OTU domain, the enzyme completely loses its activity against K48-4Ub, which indicates that L265 is a critical amino acid for the catalysis of K48-linked substrates. It is not possible to claim that the insertion of a third mutation (F82S) beside the catalytic Cys is responsible for the inactivity of F82S/F182S/L265P variant, since F182S/L265P variant is intrinsically inactive, however, the third mutation caused the formation of a large amount of inclusion bodies with F82S/F182S/L265P OtuLi in bacteria. The formation of inclusion bodies may be a consequence of the drastic changes in the protein structure leading to an inefficient misfolding caused by the coordinated action of the three mutations.

Cytolocalization assays showed that OtuLi is sorely present in a region near kinetoplast, especially in parasites with shorter flagellum or rounded-shape cell bodies. It was previously shown that *otubain* gene in *L. infantum* is differentially regulated after generation of axenic amastigote forms treated at pH 4.5, being 1.8-fold upregulated in these forms (Alcolea et al., 2010), indeed, the cellular localization of OtuLi in amastigote forms has to be performed to assert this assumption.

Previous studies suggested intracellular accumulation of lipid droplet in cells infected or stimulated with *Leishmania* (Rabhi et al., 2016) could help triggering cytokine signal (Bozza et al., 2007). Knowing that otubains have been implicated in the immunological events, we aimed to investigate the role of rOtuLi in modulation of inflammatory parameters of murine macrophages. Our data suggest that rOtuLi is capable of modulating immune and inflammatory response in murine peritoneal macrophages, since this enzyme induced lipid droplet biogenesis, an important intracellular marker of inflammation, as well as IL-6 and TNF-α secretion, two proinflammatory cytokines. However, the question whether OtuLi can be secreted by the parasite when it is inside the phagolysosome remains unanswered and needs to be further addressed. The sequence analyses of OtuLi did not show a peptide signal, but this enzyme could be secreted by non-classical routes, such as exocytosis of lysosomal compartment (McConville et al., 2002).

In summary, we identified a novel otubain-like from *L. infantum* with deubiquitinating activity located along the cytoplasm from promastigote forms and our results suggest that this enzyme could trigger inflammatory response *in vitro*. This study was also relevant to initiate future studies to elucidate the functional role of deubiquitinating enzymes with the intention to describe the deubiquitination process and address its importance in the establishment of infection by these parasites.

## Author contributions

CA and IB designed the study and wrote the paper. FM and BG designed and constructed vectors for expression of wild type and mutant proteins. JP and CA purified and performed enzyme assays. RC and KM performed the immunology assays. DN performed the protein structural study. JS and PG produced OtuLi mouse anti-serum and supported the cytolocalization analyses. All authors analyzed the results and approved the final version of the manuscript.

## Funding

This work was supported by INCT (Institutos Nacionais de Ciência e Tecnologia) grant number: 465771/2014-9, CAPES (Coordenação de Aperfeiçoamento de Pessoal de Nível Superior) - COFECUB grant number: 723/11, CNPq (Conselho Nacional de Ciência e Tecnologia) grant number: 303675/2015-2 and 307186/2013-0. FAPDF (Fundação de Apoio à Pesquisa do Distrito Federal) grant numbers: 193.001.076/2015 and 193.000.822/2015. MCT/CNPq/FNDCT/FAPs/MEC/CAPES/PRO-CENTRO-OESTE. FINEP and by the an international mobility grant from IDEX Sorbonne Universités (SUPER).

### Conflict of interest statement

The authors declare that the research was conducted in the absence of any commercial or financial relationships that could be construed as a potential conflict of interest.
